# The complete mitochondrial genome of a marbled eelpout *Lycodes raridens* (Perciformes: Zoarcidae)

**DOI:** 10.1080/23802359.2019.1688698

**Published:** 2019-11-13

**Authors:** Sang-Hwa Lee, Chung-Bae Kang, Myung-Hwa Shin, Sang-Hui Lee, Moongeun Yoon, Hyung Jun Kim

**Affiliations:** National Marine Biodiversity Institute of Korea, Seocheon, Republic of Korea

**Keywords:** Lycodes raridens, complete mitogenome, phylogenetic analysis

## Abstract

In this study, the complete mitochondrial genome of the marbled eelpout, *Lycodes raridens* Taranetz & Andriashev, 1937 was sequenced using the primer walking method. The mitogenome was 16,569 bp in length and encoded with 13 protein-coding genes, 22 transfer RNA genes, two ribosomal RNA genes, and one Non-Coding Region. The overall nucleotide composition of *L. raridens* is 25.5%, 25.3%, 18.7%, and 30.5% for A, T, G, and C, respectively. Phylogenetic analysis using the ML method showed that *L. raridens* was clustered into one branch with *L. ygreknotatus* and *L. toyamensis*.

The family Zoarcidae Swainson, 1839 is the most diverse family of order Perciformes and is widely distributed in temperate to polar regions, including the North Pacific, Arctic, and Atlantic oceans (Anderson and Fedorov 2004). Eelpouts, more than 300 species from 60 genera in the family Zoarcidae, occur in the continental shelf and bathyal zone, and play a crucial role in the bottom communities (Nelson 1994; Balanov et al. 2006; Zheng et al. 2019). In this study, we provide the complete mitochondrial genome sequence of a marbled eelpout, *Lycodes raridens* Taranetz & Andriashev, 1937 for the first time.

A sample of *L. raridens* was collected from the Antarctic Ocean, southwest of the Atlantic (65° 8′ 24.00″ S, 113° 56′ 15.00″ E) on February 2, 2017, and the voucher specimen was deposited at the National Marine Biodiversity Institute of Korea (MABIK 0016001). Total genomic DNA was extracted from the muscle tissue using the DNeay Blood & Tissue Kit (Qiagen, Hilden, Germany). Then the complete mitogenome sequences of *L. raridens* were amplified using designed five specific primer sets for long-PCR and sequenced by the primer walking method. The sequences were assembled using Geneious v9 (Kearse et al. 2012) and annotated using the MITOS (Bernt et al. 2013) web servers. Finally, the mitogenome was confirmed in comparison with two *Lycodes* species sequences, *L. ygreknotatus* and *L. toyamensis* which previously reported the complete mitogenome sequences and was deposited in the GenBank under accession number MN604279.

The complete mitochondrial genome of *L. raridens* is 16,569 bp in length and comprises 13 protein-coding genes (PCGs), 22 transfer RNA genes (tRNAs), two ribosomal RNA genes (rRNAs), and one Non-Coding Region (NCR). The mitochondrial gene arrangement of *L. raridens* was congruent with that observed in the genus *Lycodes* species. The base composition of the entire mitogenome was 25.5% for A, 25.3% for T, 18.7% for G, and 30.5% for C, respectively. Twenty-eight genes (12 PCGs, 2 rRNAs, and 14 tRNAs) were encoded on the heavy strand (H-strand), while nine genes (one PCG and eight tRNAs) were encoded on the light strand (L-strand). Except for COI and ND3, which were initiated with GTG and ATA, respectively, most of the protein-coding genes of *L. raridens* were ATG. TAA (COI, COIII, ATP6, ATP8, ND1–2, and ND4L) and TAG (ND3 and ND5–6) were used as stop codons and three PCGs (COII, CYTB, and ND4) were terminated with incomplete stop codon T, to avoid overlapping between the genes.

To confirm the phylogenetic position of *L. raridens*, other 12 fish species belonging to seven families were used (Figure 1). Phylogenetic trees were estimated based on the concatenated data set of 13 PCGs using maximum likelihood method with GTR model in MEGA7 (Kumar et al. 2016). The bootstrap values were calculated from 1,000 replicates. In Tree, *L. raridens* was clustered with its congeneric species *L. ygreknotatus* and *L. toyamensis*, and the three *Lycodes* species had the closest relationship with the family Cottidae. Our results would be valuable for evolutionary studies in the family of Zoarcidae.

**Figure 1.**
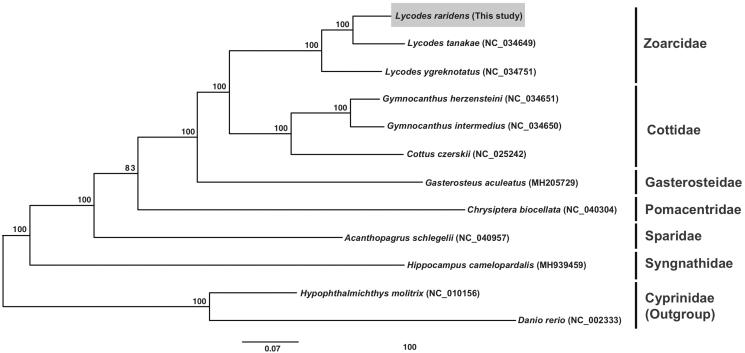
Molecular phylogeny of *Lycodes raridens* and the other fish species using concatenated 13 PCGs nucleotide dataset. The phylogenetic tree is constructed by maximum likelihood (ML) method based on 12 mitogenome sequences including *L. raridens* (This study). Bootstrap replicates were performed 1,000 times.
